# Fabrication of an In Situ pH-Responsive Raloxifene-Loaded Invasome Hydrogel for Breast Cancer Management: In Vitro and In Vivo Evaluation

**DOI:** 10.3390/ph17111518

**Published:** 2024-11-11

**Authors:** Hanan O. Farouk, Marwa M. Nagib, Amr Gamal Fouad, Demiana M. Naguib, Sherif Faysal Abdelfattah Khalil, Amany Belal, Samar F. Miski, Nisreen Khalid Aref Albezrah, Shatha Hallal Al-Ziyadi, Gi-Hui Kim, Ahmed H. E. Hassan, Kyung-Tae Lee, Doaa S. Hamad

**Affiliations:** 1 Department of Pharmaceutics, Faculty of Pharmacy, Nahda University, Beni-Suef 62511, Egypt; hanan.osman@nub.edu.eg (H.O.F.); demiana.monier@nub.edu.eg (D.M.N.); 2 Department of Pharmacology and Toxicology, Faculty of Pharmacy, Misr International University, Cairo 11435, Egypt; marwa.naguib@miuegypt.edu.eg; 3 Department of Pharmaceutics and Industrial Pharmacy, Faculty of Pharmacy, Beni-Suef University, Beni-Suef 62511, Egypt; 4 Pharmacology Department, Faculty of Medicine, Beni-Suef University, Beni-Suef 62511, Egypt; sherif.khalil@med.bsu.edu.eg; 5 Department of Pharmaceutical Chemistry, College of Pharmacy, Taif University, Taif 21944, Saudi Arabia; a.belal@tu.edu.sa; 6 Pharmacology and Toxicology Department, College of Pharmacy, Taibah University, Medina 42278, Saudi Arabia; smiski@taibahu.edu.sa; 7 Department of Obstetric & Gynecology, College of Medicine, Taif University, Taif 21944, Saudi Arabia; dr.nisreen@tu.edu.sa (N.K.A.A.); shatha.h@tu.edu.sa (S.H.A.-Z.); 8 Department of Pharmaceutical Biochemistry, College of Pharmacy, Kyung Hee University, Seoul 02447, Republic of Korea; gihi0118@khu.ac.kr; 9 Department of Fundamental Pharmaceutical Science, Graduate School, Kyung Hee University, Seoul 02447, Republic of Korea; 10 Department of Medicinal Chemistry, Faculty of Pharmacy, Mansoura University, Mansoura 35516, Egypt; 11 Department of Pharmaceutics and Pharmaceutical Technology, Faculty of Pharmacy, Nile Valley University, Fayoum 63518, Egypt; doaa.qarni@nv.edu.eg

**Keywords:** breast cancer, raloxifene, invasomes, bioavailability, chitosan, targeting

## Abstract

Background/Objectives: Raloxifene (RLF) is a therapeutic option for invasive breast cancer because it blocks estrogen receptors selectively. Low solubility, limited targeting, first-pass action, and poor absorption are some of the challenges that make RLF in oral form less effective. This study aimed to create an intra-tumoral in situ pH-responsive formulation of RLF–invasome (IPHRLI) for breast cancer treatment, with the goals of sustaining RLF release, minimizing adverse effects, and enhancing solubility, bioavailability, targeting, and effectiveness. Methods: Numerous RLF–invasome formulations were optimized using design expert software (version 12.0.6.0, StatEase Inc., Minneapolis, MN, USA). Integrating an optimal formulation with an amalgam of chitosan and glyceryl monooleate resulted in the IPHRLI formulation. In vivo testing of the IPHRLI formulation was conducted utilizing the Ehrlich cancer model. Results: Requirements for an optimum RLF–invasome formulation were met by a mixture of phospholipids (2.46%), ethanol (2.84%), and cineole (0.5%). The IPHRLI formulation substantially sustained its release by 75.41% after 8 h relative to free RLF. The bioavailability of intra-tumoral IPHRLI was substantially raised by 4.07-fold compared to oral free RLF. Histopathological and tumor volume analyses of intra-tumoral IPHRLI confirmed its efficacy and targeting effect. Conclusions: the intra-tumoral administration of the IPHRLI formulation may provide a potential strategy for breast cancer management.

## 1. Introduction

Breast cancer (BC) is a malignant disease that develops when cells in the breast multiply uncontrollably [1,2]. It claimed 670,000 lives worldwide in 2022 [1,2]. The prevalence of BC has experienced a yearly growth rate of 0.6%, posing a significant challenge to public health [3,4]. An estimated 250,000 American women will receive a breast cancer diagnosis every year [5]. Inhibiting the activity of the estrogen receptor can reduce the risk of breast cancer progression [6,7]. One major class of breast cancer medications is estrogen receptor antagonists [8,9]. Raloxifene (RLF) is a therapeutic option for invasive breast cancer because it selectively blocks estrogen receptors [8,9,10]. RLF is a worthy candidate for usage as a first-line medication in chemoprophylaxis because of its low risk of thrombosis and lack of association with endometrial cancer [11]. The challenges that make RLF in oral form less effective are its low solubility, limited targeting, first-pass action, and poor absorption [12,13].

In order to address these issues, an intra-tumoral pH-responsive in situ formulation of RLF–invasome (IPHRLI) has been fabricated with the goals of sustaining RLF release, enhancing the solubility, bioavailability, targeting, and effectiveness of RLF while also reducing its toxicity as a potential breast cancer therapy. The intra-/peri-tumor drug delivery (ITDD) is an intriguing alternative to breast cancer treatment. It has the potential to improve localization while reducing systemic toxicity [14,15,16]. The benefits of ITDD include targeting drug delivery to tumor cells and achieving the necessary therapeutic concentration of medicines at the tumor site [14,15,16]. Additionally, ITDD can minimize systemic side effects by dose reduction and eliminate frequent administrations of drugs [17,18]. The in situ pH-responsive hydrogel might help drugs be absorbed better by making them stay in the body longer and exposing tumors to RLF for longer by keeping them released [19,20]. The pH-responsive characteristics of chitosan and glyceryl monooleate have been used in numerous studies [17,18,19,20]. In acidic solutions, chitosan stays liquid; however, when it comes into contact with alkaline or biological pH, it loses its charge and turns into thick gels [20]. Adding extra water to glyceryl monooleate can form gels of high viscosity [21]. Both polymers have mucoadhesive properties and are safe, non-toxic, and biodegradable [17,18,19,20]. Glyceryl monooleate and chitosan work together synergistically to target and sustain medicines [17,18,19,20].

An invasome formulation is a kind of nanoparticulate vesicle that was created to increase a drug’s bioavailability and efficacy [22,23]. Invasomes are made up of liposomal vesicles that contain ethanol and terpenes, which are substances that increase solubility [24,25,26]. Small particle sizes of invasomes improve medication absorption. By utilizing these carriers, medications can be precisely targeted to particular tissues, and their release can be sustained, leading to a reduction in side effects [27,28].

As far as we know, no research has looked into using an intra-tumoral IPHRLI as a possible breast cancer treatment with the aims of maintaining RLF release, reducing side effects, and improving solubility, bioavailability, targeting, and effectiveness. Numerous RLF–invasome formulations were optimized using design expert software (version 12.0.6.0, StatEase Inc., Minneapolis, MN, USA). After in vitro testing, the IPHRLI formulation was made by combining an optimum formulation with a mix of chitosan and glyceryl monooleate. We conducted in vivo testing of the IPHRLI formulation using the Ehrlich cancer model.

## 2. Results and Discussion

### 2.1. Preliminary Study

Dragicevic et al. introduced invasomes, a new generation of liposomes with improved physico-chemical properties, to the market [29,30,31]. Invasomes are unique vesicles that are both flexible and filled with a combination of phospholipids, terpenes, cholesterol, and ethanol. Phospholipids and cholesterol play an important role in invasome processes such as bilayer formation, edge activation, and vesicle stiffness [27,28,32]. Cineole and ethanol enable the lipophilic RLF to dissolve and incorporate into invasomes [22,23]. After reviewing the relevant literature, we settled on the dependent and independent variables, as shown in Table 1. Several studies have shown that invasome EE and VS are significantly affected by different concentrations of phospholipids, ethanol, and terpenes [22,23,28,33]. Similarly, Kamran et al. demonstrated successful optimization of olmesartan-loaded invasomes by controlling for phospholipids, ethanol, and β-citronellene concentrations [33].

A literature review and preliminary experiments informed the choice of independent variable concentrations [22,23,25,28,34]. Phospholipid concentrations in invasomal formulations should be in range of 1–3%, as demonstrated in pre-formulation investigations. This is due to the fact that an increase in phospholipid concentration above 3% considerably raised the VS of invasomes. The particle aggregation rate and creation of bigger nanoparticles may be enhanced by an increase in VS, which may be associated with an increase in viscosity and mass transfer resistance. Following the lead of Ahmed et al., we found consistent results [24].

Invasomal formulations should have ethanol concentrations in a range of 1–5%. Due to the increased solubility of phospholipids in highly concentrated ethanol (>5%), the invasomal membrane will become more permeable, resulting in a notable decrease in EE. Invasomal formulations should have cineole concentrations in a range of 0.5–1.5%. This is due to the fact that an increase in cineole concentrations considerably lowered the EE of invasomes due to their ability to alter the usual shape of the vesicle membrane, making it more flexible. This finding is in line with what Awan et al. have already reported [30]. Based on these findings, the levels of ethanol and phospholipids were determined to be 1–5%, while the level of cineole was determined to be 0.5–1.5%. A Box–Behnken design was then adopted due to its simplicity and ability to minimize runs. Table 2 displays the outcomes of a design matrix that was created using the design expert software.

### 2.2. Characterization and Optimization of RLI Formulation

#### 2.2.1. Design Expert Software

The results of the design expert program indicated that the quadratic model, with its high order, considerable additional terms, and lack of aliasing, was the optimal choice for VS and EE. The model was deemed significant, with a *p*-value < 0.05, insignificant lack of fit, and an F-value of 1377.83 and 1293.73 for VS and EE, respectively. The “adjusted R^2^” and “predicted R^2^” for VS and EE were reasonably close, with a discrepancy of less than 0.2.

With an adequate precision of 131.974 and 128.241 for VS and EE, respectively, this model was able to successfully explore the design space, indicating a sufficient signal. Considering all experiments in Table 2, the VS and EE values showed ratios of 2.5 and 1.5, respectively, suggesting that no transformation was necessary. The need for a transformation is typically signaled by a ratio higher than 10.

**Figure 1:**
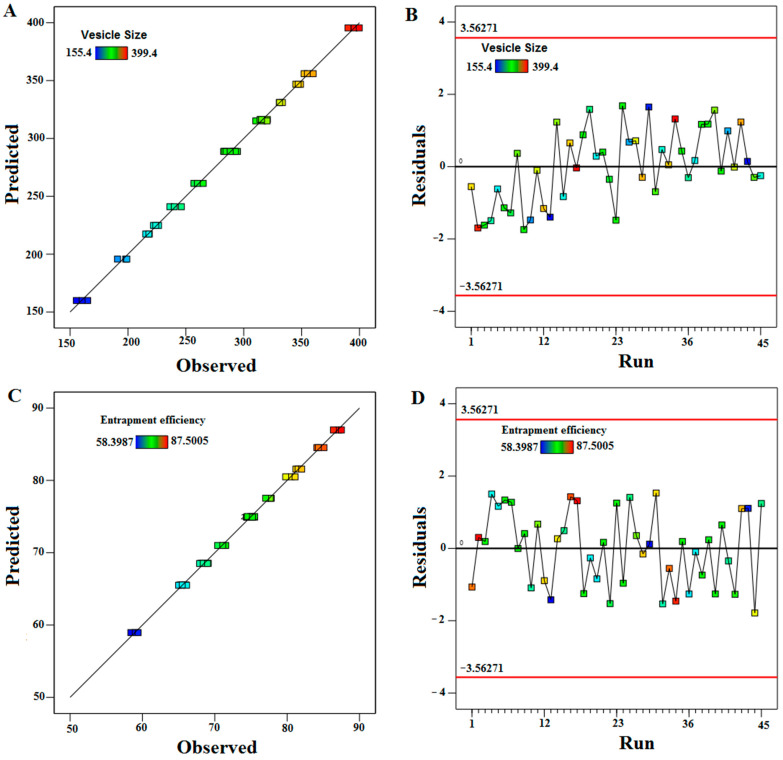
(**A**,**C**) Correlation between observed and predicted values; (**B**,**D**) correlation between residual and run for vesicle size and entrapment efficiency.

The predicted and observed values of VS and EE are quantitatively compared in Figure 1A,C and Table 2. The results showed that they are highly correlated. The experimental run order is plotted against the residuals in Figure 1B,D. During the experiment, it looks for hidden factors that could have affected the result. Figure 1B,D displays a random scatter, which verifies the strong association between the expected and observed outcomes.

#### 2.2.2. Vesicle Size

**Figure 2:**
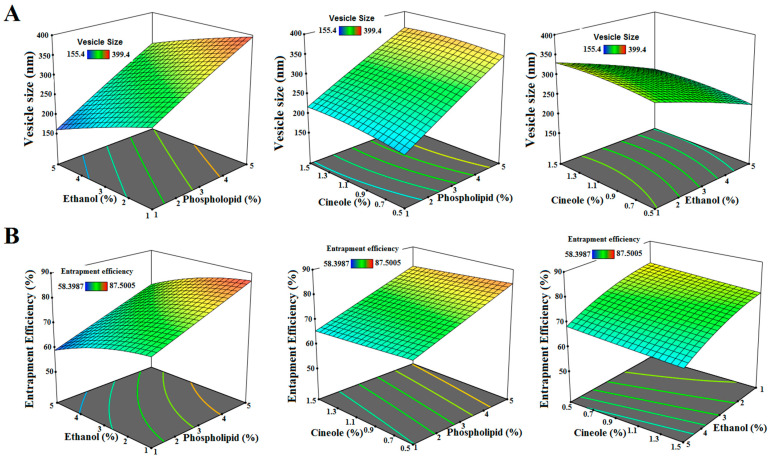
Three-dimensional charts showing the effects of factors on (**A**) vesicle size and (**B**) entrapment efficiency.

For the purpose of evaluating the effects of factors on VS and EE, the design expert software offers three-dimensional charts (Figure 2). Equations (1) and (2), using coded factors, are provided by design expert software. These equations can be used to estimate the response for given levels of each factor. The 3D plot (Figure 2A) demonstrates a considerable (*p*-value < 0.05) direct correlation between the VS of RLI and the concentrations of phospholipids and cineole while showing a considerable (*p*-value < 0.05) inverse correlation with ethanol concentration. According to Table 2, the formulations with the highest percentage of phospholipids (5%), RLI1, and RLI2 had a bigger VS than the formulations with the lowest percentage of phospholipids (1%), RLI10, and RLI7, respectively. The particle aggregation rate and creation of bigger nanoparticles may be enhanced by an increase in VS, which may be associated with an increase in viscosity and mass transfer resistance. Following the lead of Teaima et al. we found consistent results [34].

In comparison to formulations RLI4 and RLI15, which included 5% ethanol, the VS was bigger in formulations RLI11 and RLI14, which contained 1% ethanol. As the concentration of ethanol increased, the vesicles shrank due to a change in their net charge and a decreasing membrane thickness. These results concur with those of a prior study conducted by Abou-Taleb et al. [35]. In comparison to formulations RLI10 and RLI1, which included 0.5% cineole, the VS was bigger in formulations RLI5 and RLI12, which contained 1.5% cineole. Because a greater membrane surface area was required for packing, larger invasomes were produced as a result of disrupted lipid bilayer packing caused by increased cineole concentration. Similar findings were noted by El-Tokhy et al. [25].

Y_1_ (VS) = 288.74 + 72.4X_1_ − 45.4X_2_ + 7.71X_3_ + 5.22X_1_X_2_ − 3.13X_1_X_3_ + 0.383X_2_X_3_ − 2.56X_1_^2^ − 3.24X_2_^2^ − 7.18X_3_^2^ (1)

#### 2.2.3. Entrapment Efficiency

According to the 3D plot (Figure 2B), the EE of RLI is considerably (*p*-value < 0.05) positively correlated with phospholipid concentration and considerably (*p*-value < 0.05) negatively correlated with ethanol and cineole concentrations. Table 2 demonstrates that formulations RLI8 and RLI12, which had a phospholipid content of 5%, had a higher EE compared to formulations RLI13 and RLI5, which had a lower phospholipid content of 1%. An elevated phospholipid concentration promotes the development of robust and cohesive layers that envelop the drug. Following the lead of Teaima et al. we found consistent results [34].

RLI2 and RLI7, which contained 1% ethanol, had a lower EE compared to RLI8 and RLI13, which contained 5% ethanol, respectively. An elevated ethanol concentration resulted in the rupture of the vesicle membrane, facilitating the leakage of medicines from the vesicle. These results concur with those of a prior study conducted by Kamran et al. [33]. RLI1 and RLI10, which contained 0.5% cineole, had a higher EE compared to RLI12 and RLI5, which contained 1.5% cineole, respectively. An increase in cineole content may reduce the likelihood of drug entrapment by increasing the permeability and changing the regular structure of the vesicle membrane [25,36]. Conforming to the findings of Abou-Taleb et al., similar results were obtained [35].

Y_2_ (EE) = 74.92 + 8.0X_1_ − 6.0X_2_ − 1.49X_3_ + 0.017X_1_X_2_ + 0.0073X_1_X_3_ − 0.0109X_2_X_3_ + 0.049X_1_^2^ − 1.99X_2_^2^ + 0.0737X_3_^2^ (2)

#### 2.2.4. Optimization of RLI Formulation

The optimum RLI formulation was selected by using numerical point prediction. Optimum RLI formulation requirements were met by the formulation composition with concentrations of phospholipids (2.46%), ethanol (2.84%), and cineole (0.5%). The optimum RLI formulation had an observed VS of 253.8 ± 5.21 nm and an EE of 74.2 ± 0.61%. It was concluded that the optimum RLI formulation was reasonable and dependable because the experimental values of VS and EE produced by it were in accordance with the predicted values of VS (257 nm) and EE (74.8%), respectively, produced by design expert software.

### 2.3. Characterization of Optimum RLI Formulation

#### 2.3.1. Size Distribution and Zeta Potential

**Figure 3:**
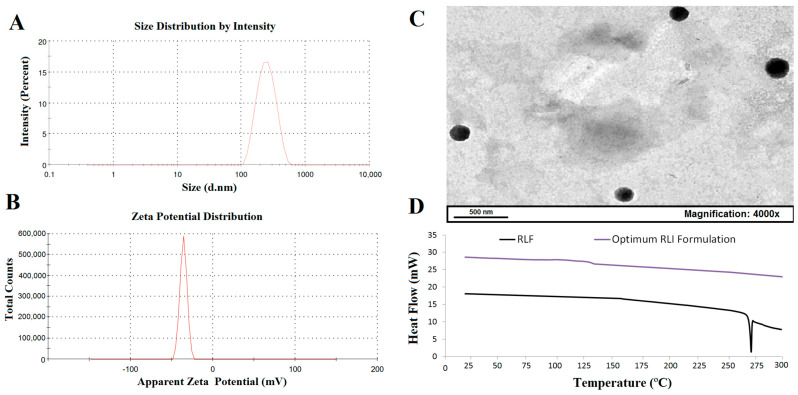
(**A**) PDI; (**B**) zeta potential; (**C**) surface morphology, where photographs were taken at 4000× magnification and scale bar = 500 nm; and (**D**) DSC thermograms of optimum RLI formulation.

A PDI value below 0.2 suggests that the formulations are mostly uniformly distributed [37,38]. As illustrated in Figure 3A, an optimum RLI had a PDI of 0.195, indicating a homogeneous and dispersed vesicle. A negative zeta potential suggests that the formulations are mostly stable [37,38]. As illustrated in Figure 3B, an optimum RLI had a zeta potential of (−) 40.4, indicating its de-aggregation and physical stability.

#### 2.3.2. Morphology Evaluation

Using TEM, the shape of the optimum RLI formulation revealed spherical vesicles that were nano-sized, homogenous, non-aggregated, and identifiable, as illustrated by Figure 3C.

#### 2.3.3. Crystallinity Evaluation

Figure 3D illustrates an intriguing endothermic peak at 269.9 °C, indicative of RLF’s melting temperature. The optimum RLI formulation did not show this peak in its DSC, which would indicate that RLF was in an amorphous form and entirely embedded in invasomes.

### 2.4. Preparation and Characterization of Optimum IPHRLI Formulation

#### 2.4.1. Preparation of IPHRLI Formulation

The long-term sustainability of drugs can be enhanced by using in situ polymers that possess acceptable rheological and mucoadhesive characteristics [39,40]. A literature review and preliminary experiments informed the choice of in situ polymer concentrations [17,18,20,41,42]. Chitosan is an in situ polymer that is compatible and does not cause toxicity [17,18]. It might be a better choice as a carrier for making a pH-responsive hydrogel, since chitosan solutions retain their liquid state at a low pH and gel state at a high pH [20,42]. The combination of chitosan with glyceryl monooleate results in a thick gel [20,42]. A literature review and preliminary experiments informed the choice of 0.67% *w*/*v* of chitosan and 0.27% *w*/*v* of glyceryl monooleate. Following the lead of Salem et al., we found consistent results [17,18].

#### 2.4.2. pH Evaluation

The ideal pH range for the formulations is between 5.5 and 7.4 [20]. The IPHRLI and FRIPH formulations were determined to have pH values of 5.6 ± 0.2 and 5.8 ± 0.3, respectively, which are deemed adequate. The formulations maintained their liquid state at both room temperature and the pH where they were created.

#### 2.4.3. Gelation Studies

A moderate score (++) was achieved by the IPHRLI and FRIPH formulations. The findings demonstrated that the IPHRLI formulation effectively provided RLF with a sustained release.

#### 2.4.4. Viscosity Study

**Figure 4:**
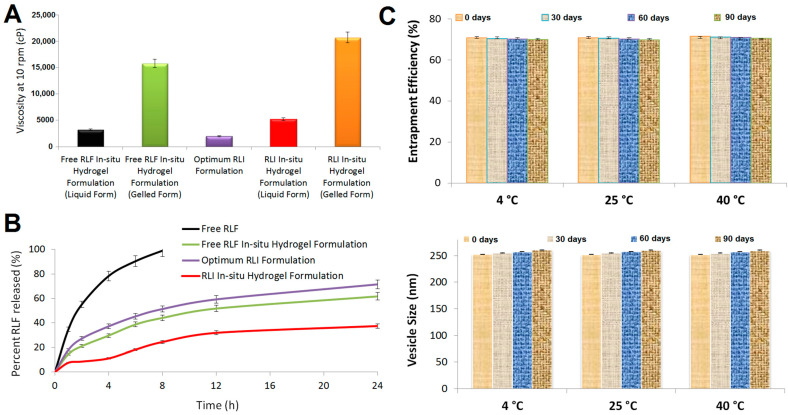
(**A**) Viscosity of IPHRLI formulation compared to FRIPH formulation and optimum RLI formulation; (**B**) release profile of IPHRLI formulation compared to free RLF and optimum RLI formulation; (**C**) impact of different storage temperatures on IPHRLI formulation’s entrapment efficacy and vesicle size.

Figure 4A shows that the IPHRLI formulation substantially increased the viscosity of the optimum RLI (*p*-value < 0.05). Glyceryl monooleate and chitosan’s synergistic mucoadhesive activity produced these results. Both the liquid and gelled forms of the IPHRLI formulation had substantially greater viscosities than the FRIPH formulation (*p*-value < 0.05) because invasomes’ phospholipids cause the creation of thick bilayers.

#### 2.4.5. In Vitro Release Kinetics Study

Figure 4B shows that all of the RLF was released from the free RLF suspension within 8 h. The sustained release pattern of the IPHRLI formulation was indicated by a 75.41%, 47.68%, and 33.91% reduction in the percentage of RLF compared to free RLF, the optimum RLI formulation, and FRIPH formulation, respectively, which was statistically significant (*p*-value < 0.05). The mucoadhesive activity and high viscosity of invasomes, glyceryl monooleate, and chitosan explain the slow release of the IPHRLI formulation. The MDT provided by the DDSolver tool was used to validate these results [43]. Its 7.13 MDT verified the IPHRLI formulation’s sustainability.

In order to determine how RLF released from the IPHRLI formulation, the DDSolver tool was utilized [27,28]. The Korsmeyer–Peppas model offered the best fit for the IPHRLI formulation, as indicated by its highest R2 and MSC values and lowest AIC. The computed “*n*” was recorded as 0.725, indicating that the release of RLF from the IPHRLI formulation was caused by non-Fickian diffusion. A high *f*1 value of 80.54 and a low *f*2 value of 12.92 were indicative of a significant variation between the IPHRLI formulation and free RLF profiles.

#### 2.4.6. Stability Study

The IPHRLI formulation remained constant at 4 °C, 25 °C, and 40 °C, as shown in Figure 4C, due to the absence of a significant change (*p*-value > 0.05) in entrapment efficiency and vesicle size after three months.

### 2.5. In Vivo Evaluation of IPHRLI Formulation

#### 2.5.1. Bioavailability Study

**Figure 5:**
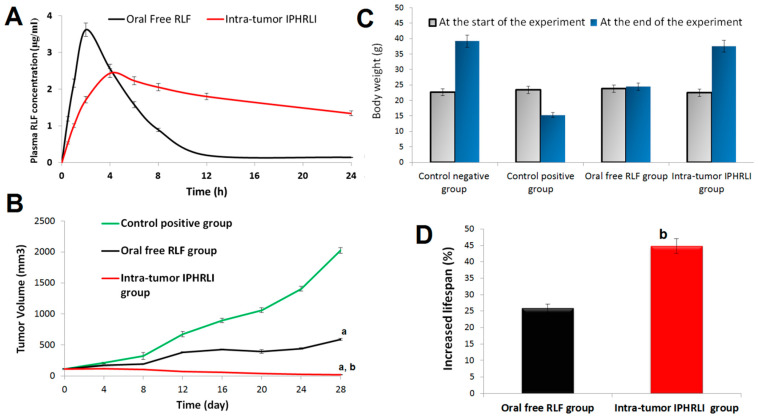
(**A**) RLF plasma concentration–time profile between intra-tumor IPHRLI formulation and oral free RLF; (**B**) variation in tumor volume between control positive group and groups treated with intra-tumor IPHRLI formulation or oral free RLF; (**C**) variation in body weight between control positive group and groups treated with intra-tumor IPHRLI formulation or oral free RLF; (**D**) intra-tumor IPHRLI formulation’s RLF concentration in liver and tumor compared to oral free RLF. ^a^ Significance when compared to control positive group, with *p*-value < 0.05; ^b^ Significance when compared to oral free RLF group, with *p*-value of <0.05.

By tracking its plasma levels using HPLC, the bioavailability of oral free RLF and intra-tumor IPHRLI was assessed. Figure 5A shows the results of a non-compartmental approach that measured the plasma drug concentration versus time for both oral free RLF and intra-tumor IPHRLI. The Cmax, Tmax, t_0.5_, MRT, and AUC were found with the help of pK solver software [44], as shown in Table 3. Compared with oral free RLF, the intra-tumor administration of IPHRLI caused a 32.77% decrease in Cmax, which was statistically significant (*p*-value < 0.05). This means that there was less unwanted RLF concentration, which means there were fewer side effects. Sustainability metrics used to assess the formulation include Tmax, MRT, and t_0.5_ [45,46]. Compared with oral free RLF, the intra-tumor administration of IPHRLI caused a 2-, 5.8-, and 5.49-fold increment in Tmax, MRT, and t_0.5_, which was statistically significant (*p*-value < 0.05). These results reported a slow plasma drug clearance and hence, no frequent dosing was required and less adverse effects occurred. These results confirmed the sustainability of IPHRLI, as reported by an in vitro release study. There was a 4.07-fold enhancement in relative bioavailability between the oral free RLF and intra-tumor IPHRLI, as shown in Table 3. Due to its increased surface area, the nano-sized IPHRLI formulation may have a higher absorption capacity. The presence of cineole and ethanol enhanced the drug solubility.

#### 2.5.2. Antitumor Activity Study

Body weight, mortality rate, tumor volume, and the histological features of tumors were some of the criteria monitored to confirm that breast cancer was successfully progressing [47,48,49].

**Figure 6:**
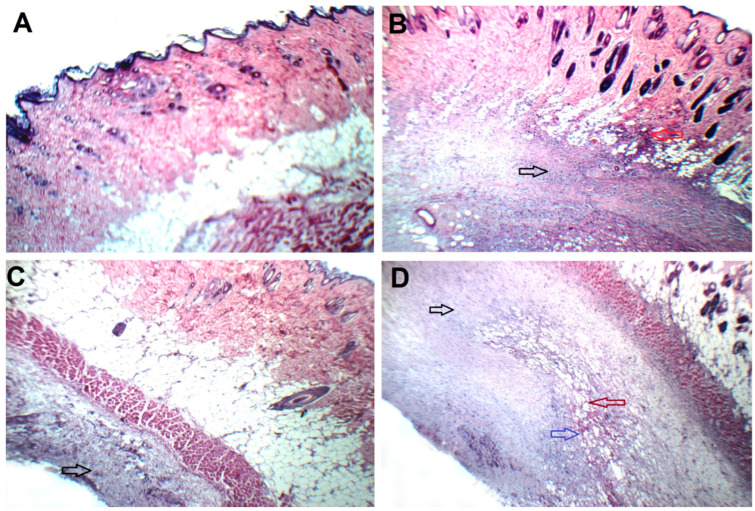
Histopathology findings of tumor of (**A**) control negative group, (**B**) control positive group, (**C**) oral free RLF group, and (**D**) intra-tumor IPHRLI formulation group. Black arrow: hypercellular tumor; red arrow: infiltrating surrounding fat; blue arrow: necrosis area; violet arrow: cyst formation.

There was a 18.17-fold increment (*p*-value < 0.05) in tumor volume and a 34.66% reduction (*p*-value < 0.05) in the body weight of the control positive group between the end and start of the experiment, as shown in Table 4 and Figure 5B,C. There was a 60.94% reduction (*p*-value < 0.05) in the body weight and a 31.07% increment (*p*-value < 0.05) in the mortality rate between the control positive group and the control negative group. Histopathology findings (Figure 6B) of the control positive group displayed a large hypercellular tumor (black arrow) composed of large mildly pleomorphic rounded and spindle cells with scattered mitotic figures, congested blood vessels, and infiltrating surrounding fat (red arrow). Evidence like this proves that the experimental breast cancer model was effectively administered to the mice.

There was a 70.95% reduction (*p*-value < 0.05) in the tumor volume, a 59.80% increment (*p*-value < 0.05) in the body weight, and a 25.86% reduction (*p*-value < 0.05) in the mortality rate between the oral free RLF and the control positive group, as shown in Table 4 and Figure 5B–D, respectively. There was a 1.03-fold increment (*p*-value < 0.05) in the body weight of the oral free RLF group between the end and start of the experiment, as shown in Figure 5C. Histopathological findings (Figure 6C) of the oral free RLF group displayed a little hypocellular tumor (black arrow) composed of mildly pleomorphic rounded cells with scattered apoptosis and without infiltrating muscles.

**Figure 7:**
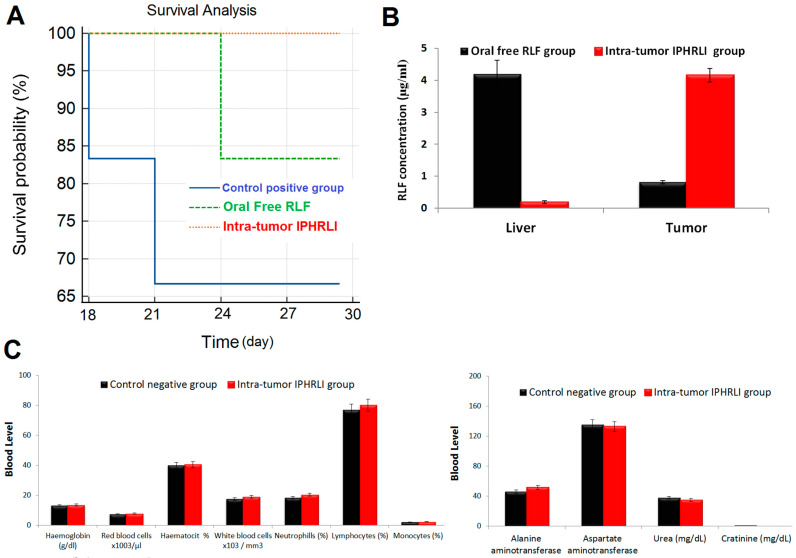
(**A**) Kaplan–Meier survival curve of oral free RLF, intra-tumor IPHRLI formulation, and control positive group; (**B**) liver and tumor concentration of RLF for intra-tumor IPHRLI formulation and oral free RLF groups; (**C**) biochemical and hematological parameters of intra-tumor IPHRLI formulation group compared to control negative group.

There was an 80.40% reduction (*p*-value < 0.05) in tumor volume and a 1.67-fold increment (*p*-value < 0.05) in the body weight of the intra-tumor IPHRLI formulation group between the end and start of the experiment, as shown in Table 4 and Figure 5B,C, respectively. There was a 98.91% reduction (*p*-value < 0.05) in the tumor volume, a 1.45-fold increment (*p*-value < 0.05) in the body weight, and a 44.83% reduction (*p*-value < 0.05) in the mortality rate between the intra-tumor IPHRLI formulation and the control positive group, as shown in Figure 5B–D, respectively. The survival time was extended with intra-tumor IPHRLI treatment, with an MST of 28 days in the IPHRLI group compared to the control positive group of 19.33 days (Figure 7A). Histopathological findings (Figure 6D) of the intra-tumor IPHRLI group revealed a tiny hypocellular tumor (black arrow) with necrosis areas (blue arrow), cysts (violet arrow), and blood vessels. This study’s results validated the efficacy of intra-tumor IPHRLI in treating mice.

There was a 96.25% reduction (*p*-value < 0.05) in the tumor volume, a 53.37% increment (*p*-value < 0.05) in the body weight, and a 15.08% reduction (*p*-value < 0.05) in the mortality rate between the intra-tumor IPHRLI formulation and the oral free RLF group, as shown in Table 4 and Figure 5B–D, respectively. According to the histological evaluation, the anti-cancer impact of intra-tumor IPHRLI was stronger than that of oral free RLF. This was attributed to the enhanced solubility and bioavailability of RLF obtained by the development of an intra-tumor IPHRLI.

#### 2.5.3. Targeting Studies

Figure 7B shows the targeting ability of the oral free RLF and intra-tumor IPHRLI formulation groups. Based on the results, it was observed that the intra-tumor IPHRLI formulation significantly (*p*-value < 0.05) increased the tumor concentration of RLF by 67.49% compared to the oral RLF. The intra-tumor IPHRLI formulation maintained a higher concentration of RLF in tumors than oral free RLF by 4.15-fold, indicating that the intra-tumor IPHRLI formulation could accumulate more RLF in tumor tissues, improving its targeting effect and anti-tumor activity.

#### 2.5.4. Toxicity Studies

Mice given an intra-tumor IPHRLI formulation did not die or exhibit any noticeable changes in behavior. Animals treated with the intra-tumor IPHRLI formulation did not have any negative effects on growth (Figure 5C); this was supported by the fact that the treatment group did not differ significantly from the control negative group in terms of body weight (*p*-value > 0.05). Animals treated with the intra-tumor IPHRLI formulation did not have any negative effects on erythropoiesis (Figure 7C); this was supported by the fact that the treatment group did not differ significantly from the control negative group in terms of hemoglobin, RBC count, or hematocrit (*p*-value > 0.05). Animals treated with the intra-tumor IPHRLI formulation did not have any negative effects on immunity (Figure 7C); this was supported by the fact that the treatment group did not differ significantly from the control negative group in terms of white blood cells, neutrophils, monocytes, and lymphocytes (*p*-value > 0.05). Animals treated with the intra-tumor IPHRLI formulation did not have any negative effects on renal and liver functions (Figure 7C); this was supported by the fact that the treatment group did not differ significantly from the control negative group in terms of ALT, AST, urea, and creatinine (*p*-value > 0.05). Based on these results, the intra-tumor IPHRLI formulation is a safe option for breast cancer management.

## 3. Materials and Methods

### 3.1. Materials

Sigma-Aldrich, (St. Louis, MO, USA), was approached for the acquisition of ethanol, glyceryl monooleate, chitosan, phospholipids, and cineole. Only pharmaceutical-grade chemicals were used.

### 3.2. Optimization of RLF-Loaded Invasomes (RLIs)

Fifteen RLI runs were generated utilizing the Box–Behnken design as a three-factor design with three levels using design expert software [50]. The independent and dependent variables are presented in Table 1. An ANOVA was used to assess their statistical significance. To study how various parameters affect invasome vesicle size (VS) and entrapment efficiency (EE), fifteen distinct RLI formulations were prepared, as shown in Table 2.

### 3.3. Preparation and Characterization of RLI Formulation

#### 3.3.1. Preparation of RLI Formulation

Using the thin-film hydration method, fifteen different RLI formulations were fabricated [33]. The following ingredients were dissolved in a flask containing methanol/chloroform solution: phospholipids, RLF (10 mg), cholesterol (0.016 mg), and cineole, as given in Table 2. In order to achieve a thin lipid film, the organic solvent was removed under vacuum conditions using a rotary evaporator at 100 rpm until no trace of organic odor remained. To create an invasomal suspension, the lipid film was hydrated by subjecting it to 60 °C for 1 h with ethanol and phosphate-buffered saline (PBS, pH 7.4). The resulting vesicles were subjected to 30 min of ultrasonication, and the various RLI formulations were kept at 4 °C for further analysis.

#### 3.3.2. Vesicle Size (VS)

A Zetasizer device (Malvern Instruments, UK) was utilized to ascertain the VS of the various RLI formulations that were created [31]. Before the measurements were taken, a suitable volume of deionized water (9 mL) was added to each formulation sample (1 mL) and the samples were measured three times at 25 °C.

#### 3.3.3. Analysis of Entrapment Efficiency (EE)

Different RLI formulations were evaluated for their entrapment efficiency using the ultracentrifugation method [51]. The RLI formulation underwent centrifugation in a centrifuge at 15,000 rpm for duration of 2 h at a temperature of 4 °C. After collecting the supernatant, the RLF absorbance was measured in triplicate at 293 nm. The following is the equation that was used to determine the percentage of RLF that became trapped:

(3) $$\mathrm{Entrapment}\;\mathrm{Efficiency}\;(\%)=\frac{Initial\;amount\;of\;RLF\;added-amount\;of\;free\;RLF}{Initial\;amount\;of\;RLF\;added}\times 100$$

#### 3.3.4. Optimization of RLI Formulation

The numerical point prediction optimization approach used the desirability function to identify the optimum RLI formulation [50]. Maximizing EE while minimizing VS was the criterion used to choose the optimum formulation.

### 3.4. Characterization of Optimum RLI Formulation

#### 3.4.1. Size Distribution and Zeta Potential

In order to assess the level of uniformity in the size and distribution of the vesicles, we employed the polydispersity index (PDI) [52]. We also determined how stable vesicles were obtained by looking at the zeta potential [52]. Following the previously published technique of VS measurement, the zeta potential and PDI were established using the Zetasizer [31].

#### 3.4.2. Transmission Electron Microscopy (TEM)

The visualization of vesicles was achieved with the use of transmission electron microscopy (Tokyo, Japan) [53]. After placing the sample on a carbon-coated copper grid, it was stained by phosphotungestic acid and examined under a transmission electron microscope after the grid had been left to air dry completely.

#### 3.4.3. Differential Scanning Calorimetry (DSC)

Using differential scanning calorimetry (DSC, Shimadzu, Kyoto City, Japan), the invasome components and RLF were analyzed for their thermal behavior and crystallinity [27]. In a conventional aluminum pan, RLF and optimum RLI samples were compressed and subjected to heating at 10 °C/min between 20 and 300 °C, while nitrogen was continuously purged at 100 mL/min.

### 3.5. Preparation and Characterization of Optimum IPHRLI Formulation

#### 3.5.1. Preparation of IPHRLI Formulation

To fabricate the pH-responsive in situ (IPH) formulation, a mixture of chitosan and glyceryl monooleate in citric acid was obtained [18]. Drops of melted 0.27% *w*/*v* glyceryl monooleate were added to a solution of 0.67% *w*/*v* chitosan while stirring. The optimum RLI was then slowly and gently stirred into the prepared IPH formulation to generate the IPHRLI formulation. The free RLF was slowly and gently stirred into the prepared IPH formulation to generate the free RLF pH-responsive in situ (FRIPH).

#### 3.5.2. pH Evaluation

To ensure patient safety, FRIPH and IPHRLI formulations should have a pH that is stable and causes no irritation when administered [20]. The FRIPH and IPHRLI formulations were tested using a digital pH meter to determine their optimal pH range.

#### 3.5.3. Gelation Studies

To determine the rate and magnitude of gelation, we evaluated IPHRLI’s gelling capacity [19,20]. The IPHRLI and FRIPH formulation samples were mixed with PBS (pH 7.4), and the duration it took for gel formation and disintegration allowed for a visual assessment of its gelling capacity. Gel formation should occur within 60–90 s. Collapse after 1–2 h results in the lowest score (+), a moderate collapse after 3 h yields a moderate score (++), and collapse after 7–8 h yields the highest score (+++).

#### 3.5.4. Rheological Study

To determine how efficient IPHRLI and FRIPH formulations are in the body involves its viscosity [19,20]. The optimum RLI, IPHRLI, and FRIPH formulations were tested for their viscosity using a Brookfield viscometer (version DV-III ULTRA, Middleborough, MA, USA). The shear rate was set at 10 rpm in order to obtain the measurements. Three separate calculations were made at 25 °C to determine the viscosity of the optimum RLI, IPHRLI, and FRIPH formulations.

#### 3.5.5. In Vitro Release Kinetics Study

Determining the saturation solubility of RLF by an equilibrium solubility study helped in determining the sink conditions needed for release studies. An excess of RLF was poured into a glass bottle containing PBS (pH 7.4) and then agitated for 72 h in a thermostatically controlled shaker. After filtering the mixture, we used spectrophotometry at 293 nm to determine the RLF content in triplicate.

The release investigation of the free RLF suspension, optimum RLI, IPHRLI, and FRIPH formulations was conducted using the dialysis membrane method [53]. An appropriate amount of each formulation, which is equivalent to 3 mg of RLF, was used. The equipment had a release medium of PBS (pH 7.4, 50 mL) with Tween 80 (0.1% *w*/*w*). Continuous stirring at 100 rpm and a temperature of 37 ± 1 °C were used for the Hanson dissolution apparatus. Three milliliters of medium was removed from the receptor compartment and reintroduced at various intervals throughout the course of 24 h to maintain the sink state. After collecting the samples, the RLF absorbance was measured in triplicate at 293 nm and the percentage of RLF that became released after 24 h was calculated.

For the purpose of evaluating the kinetics of drug release and comparing the results to those of a reference product, DDSolver was utilized in Microsoft Excel [54]. The model selection criterion (MSC), Akaike information criterion (AIC), and correlation coefficient (R^2^) were computed to determine the best-fitting model. It was necessary to find the release exponent “*n*” of the Korsmeyer–Peppas equation in order to select the IPHRLI’s release mechanism. To pick the IPHRLI’s sustainability, the mean dissolving time (MDT) was determined [43]. To pick the IPHRLI’s difference with free RLF, the similarity (*f*2) and difference (*f*1) factors were determined [55].

#### 3.5.6. Stability Study

To assess the stability of the IPHRLI formulation, we monitored VS and EE changes for three months at 4 °C, 25 °C, and 40 °C [53].

### 3.6. In Vivo Evaluation of IPHRLI Formulation

#### 3.6.1. Procedure

The Ethics Committee of Beni-Suef University (BSU-IACUC: 024-013) gave its approval for all of the study protocols. Using standard laboratory conditions, we gathered 24 male Swiss albino mice (20–25 g) and 12 male Wistar rats (200–250 g) and housed them in a controlled environment.

#### 3.6.2. Cancer Induction and Management

The Swiss albino mice were randomly divided into four groups of six after being fed a standard diet for one week. The first group had a normal diet and functioned as a negative control. Three other groups served as experimental groups. A subcutaneous injection of the Ehrlich carcinoma cell line (0.2 mL/2–2.5 × 10^6^ cells/mouse) was administered to each animal of experimental groups in order to induce breast cancer in mice [17]. After a period of 10 days, solid Ehrlich carcinomas with average tumor volumes measuring 111.7 ± 8.6 mm^3^ emerged in every mouse. The second group served as the positive control group and maintained an intra-tumor injection of isotonic saline solution. The third group served as the free RLF group and was orally given a solution of free RLF suspension (10 mg/kg) using a needle inserted into their mouth. The fourth group served as the IPHRLI group and maintained an intra-tumor injection of the IPHRLI formulation (10 mg/kg). After one day and six days of tumor development, the mice were given two doses of each treatment for 28 days [16].

#### 3.6.3. Bioavailability Studies

Each set of six Wistar rats was randomly assigned to a different group. One group was orally given free RLF (10 mg/kg) using an oral gavage needle. The second group received an intra-tumor administration of the IPHRLI formulation at a dose of 10 mg/kg. After 24 h, 200 µL of blood was drawn using the retro-orbital plexus method and placed in EDTA tubes. It was necessary to spin the EDTA tubes at 10,000 rpm for 5 min to extract the plasma. The plasma sample was mixed with 0.2 mL of acetonitrile and following 2 min of vigorous vortex mixing, the liquid was centrifuged at 13,000 rpm. Using a previously published and revalidated HPLC method, the drug content in the plasma supernatant was assessed [56]. Achieving an isocratic separation of RLF using a mobile phase consisting of 33% buffer solution (pH 3.0) and 67% acetonitrile was conducted using a C-18 column, which had dimensions of 4.6 mm × 150 mm, at a 1 mL/min flow rate and 287 nm. Pharmacokinetic parameters were measured using the pK solver program. These parameters include Cmax, Tmax, t_0.5_, MRT, and AUC [44].

#### 3.6.4. Anti-Tumor Activity Measurement

Monitoring the tumor’s volume and body weight was employed to assess the efficiency of the therapy [17]. Two weekly assessments of tumor volume and body weight were conducted throughout the duration of the trial. A digital caliper was used for measuring the tumor volume, while a balance was employed for body weight.

(4) $$\mathrm{Tumor}\;\mathrm{volume}=\frac{{\left(width\;of\;tumour\;mass\right)}^{2}\times length\;of\;tumour\;mass}{2}$$

At the conclusion of the experiment, the survival rate of each group was monitored using a Kaplan–Meier survival analysis in MedCal software as follows [49]:

(5) $$\mathrm{Increased}\;\mathrm{lifespan}\;(\mathrm{ILS})=\left(\frac{mean\;survival\;time\;of\;treatment\;group}{mean\;survival\;time\;of\;control\;positive\;group}-1\right)\times 100$$

#### 3.6.5. Histopathology Study

All rats were injected intraperitoneally with a ketamine/xylazine mixture at the end of the trial. After, cervical dislocation was used as a method of euthanasia. In a 10% buffered formaldehyde solution, the tumors were meticulously removed and stabilized [39]. For histological studies, slices of 4–6 µm were prepared by slicing the tumors and staining them with hematoxylin and eosin [38].

#### 3.6.6. Targeting Study

In order to determine the amount of RLF that remained in each organ, both the free RLF and IPHRLI groups’ tumor and liver tissues were cut, placed in separate tubes with acetonitrile, and subjected to sonication for 30 min to ensure the complete release of RLF. To get rid of any undesirable contaminants, the mixture was spun in a centrifuge at 6000× *g* for a duration of 10 min. After separating the clear liquid, it was filtered through a membrane filter with a pore size of 0.45 μm and then evaporated. A mobile phase comprising 33% buffer solution (pH 3.0) and 67% acetonitrile was used to reconstitute the residue. Using a previously published and revalidated HPLC method, the drug content was assessed [56].

#### 3.6.7. Toxicity Studies

Throughout the investigation, we carefully observed any behavioral changes and documented the weights of the mice in both the control negative and IPHRLI groups [53]. The standard procedure was adhered to in order to measure the following parameters in both groups: hemoglobin, ALT, AST, urea, creatinine, RBC, WBC, neutrophils, lymphocytes, monocytes, and hematocrit [53].

### 3.7. Statistical Analysis

SPSS (version 22; Chicago, IL, USA) for data analysis was used. Statistical significance was determined when the *p*-value was < 0.05.

## 4. Conclusions

RLF is a therapeutic option for invasive breast cancer because it blocks estrogen receptors selectively. No studies have investigated the possibility of using an intra-tumoral IPHRLI as a potential breast cancer therapy with the goals of sustaining RLF release, minimizing adverse effects, and enhancing solubility, bioavailability, targeting, and effectiveness. There was a 98.91% reduction (*p*-value < 0.05) in the tumor volume, a 1.45-fold increment (*p*-value < 0.05) in the body weight, and a 44.83% reduction (*p*-value < 0.05) in the mortality rate between the intra-tumor IPHRLI formulation and the control positive group. The survival time was extended with the intra-tumor IPHRLI treatment. Histopathological findings validated the efficacy of intra-tumor IPHRLI in treating mice. Based on these results, the intra-tumor IPHRLI formulation is a safe option for breast cancer management.

## Figures and Tables

**Table 1:** The independent and dependent variables for RLI preparation.

Variables	Levels				
	−1		0		+1
Independent variables X_1_: phospholipid concentration (% *w*/*v*)	1		3		5
X_2_: ethanol concentration (% *v*/*v*)	1		3		5
X_3_: cineole concentration (% *v*/*v*)	0.5		1		1.5
Dependent variables					
Y_1_: vesicle size (nm)					
Y_2_: entrapment efficiency (%)					

**Table 2:** Composition of RLI formulations with design expert software-generated observed and anticipated responses.

Formulations	Independent Variables			Dependent Variables (*n* = 3)			
				Observed Value		Predicted Value	
	X_1_ (%)	X_2_ (%)	X_3_ (%)	VS (nm ± SD)	EE (% ± SD)	VS (nm ± SD)	EE (% ± SD)
F1	5	3	0.5	347 ± 2.00	84.51 ± 0.52	346.8	84.53
F2	5	1	1	395.1 ± 4.86	87.00 ± 0.56	395.5	86.98
F3	3	3	1	288.4 ± 4.69	74.80 ± 0.36	288.7	74.92
F4	3	5	1.5	240.8 ± 4.99	65.50 ± 0.55	241.0	65.51
F5	1	3	1.5	217.3 ± 1.63	65.57 ± 0.41	217.4	65.54
F6	3	3	1	289.3 ± 4.18	75.03 ± 0.45	288.7	74.92
F7	1	1	1	260.7 ± 4.04	71.00 ± 0.56	261.2	71.00
F8	5	5	1	315.7 ± 4.94	75.00 ± 0.50	315.2	75.00
F9	3	3	1	288.5 ± 5.95	74.94 ± 0.38	288.7	74.92
F10	1	3	0.5	196.0 ± 4.36	68.53 ± 0.51	195.7	68.54
F11	3	1	1.5	331.7 ± 1.48	77.50 ± 0.42	331.0	77.53
F12	5	3	1.5	355.7 ± 3.95	81.57 ± 0.41	355.9	81.57
F13	1	5	1	160.3 ± 4.90	58.94 ± 0.50	159.9	58.96
F14	3	1	0.5	316.6 ± 3.33	80.50 ± 0.66	316.4	80.49
F15	3	5	0.5	224.3 ± 2.15	68.54 ± 0.56	224.8	68.51

X_1_: phospholipid concentration; X_2_: ethanol concentration; X_3_: cineole concentration.

**Table 3:** Comparison of intra-tumor IPHRLI pharmacokinetic parameters with those of oral free RLF.

Pharmacokinetic Parameters	Intra-Tumor IPHRLI	Oral Free RLF
Cmax (µg/mL)	2.44 ± 0.19 ^a^	3.63 ± 0.30
Tmax (h)	4 ^a^	2
AUC_0-α_ (µg·h/mL)	89.44 ± 6.00 ^a^	21.97 ± 1.82
t_0.5_ (h)	24.76 ± 1.46 ^a^	4.51 ± 0.13
MRT (h)	37.19 ± 2.13 ^a^	6.42 ± 0.16
Relative bioavailability	4.07 ^a^	

Data represent the mean value ± standard deviation (SD), *n* = 6. ^a^ Significant at *p*-value less than 0.05 compared to oral free RLF.

**Table 4:** Anti-tumor activity of IPHRLI formulation.

Anti-Tumor Activity Parameters	Control Positive	Oral Free RLF	Intra-Tumor IPHRLI
Tumor volume (mm^3^) at the end of the experiment	2026.63 ± 43.99	588.81 ± 18.28	22.08 ± 2.77
Body weight (g) at the end of the experiment	15.3 ± 1.09	24.45 ± 1.56	53.37 ± 1.87
Survival time (days) at the end of the experiment	19.33	24.33	28
Increased lifespan (%)		25.86 *	44.83 *

* Related to control positive group.

## Data Availability

The present investigation includes all datasets that were produced.
